# Computed Tomography Imaging-Based Radiogenomics Analysis Reveals Hypoxia Patterns and Immunological Characteristics in Ovarian Cancer

**DOI:** 10.3389/fimmu.2022.868067

**Published:** 2022-03-28

**Authors:** Songwei Feng, Tianyi Xia, Yu Ge, Ke Zhang, Xuan Ji, Shanhui Luo, Yang Shen

**Affiliations:** ^1^ Department of Obstetrics and Gynaecology, Zhongda Hospital, School of Medicine, Southeast University, Nanjing, China; ^2^ Department of Radiology, Zhongda Hospital, School of Medicine, Southeast University, Nanjing, China; ^3^ Department of Gynaecology, The Second Affiliated Hospital of Soochow University, Soochow University, Suzhou, China

**Keywords:** radiogenomics, computed tomography, ovarian cancer, prognosis, molecular subtypes

## Abstract

**Purpose:**

The hypoxic microenvironment is involved in the tumorigenesis of ovarian cancer (OC). Therefore, we aim to develop a non-invasive radiogenomics approach to identify a hypoxia pattern with potential application in patient prognostication.

**Methods:**

Specific hypoxia-related genes (sHRGs) were identified based on RNA-seq of OC cell lines cultured with different oxygen conditions. Meanwhile, multiple hypoxia-related subtypes were identified by unsupervised consensus analysis and LASSO–Cox regression analysis. Subsequently, diversified bioinformatics algorithms were used to explore the immune microenvironment, prognosis, biological pathway alteration, and drug sensitivity among different subtypes. Finally, optimal radiogenomics biomarkers for predicting the risk status of patients were developed by machine learning algorithms.

**Results:**

One hundred forty sHRGs and three types of hypoxia-related subtypes were identified. Among them, hypoxia-cluster-B, gene-cluster-B, and high-risk subtypes had poor survival outcomes. The subtypes were closely related to each other, and hypoxia-cluster-B and gene-cluster-B had higher hypoxia risk scores. Notably, the low-risk subtype had an active immune microenvironment and may benefit from immunotherapy. Finally, a four-feature radiogenomics model was constructed to reveal hypoxia risk status, and the model achieved area under the curve (AUC) values of 0.900 and 0.703 for the training and testing cohorts, respectively.

**Conclusion:**

As a non-invasive approach, computed tomography-based radiogenomics biomarkers may enable the pretreatment prediction of the hypoxia pattern, prognosis, therapeutic effect, and immune microenvironment in patients with OC.

## Introduction

Ovarian cancer (OC) has the highest mortality rate among gynecologic cancers. Surgery and platinum-based chemotherapy are the mainstays of care for individuals with OC (1). Meanwhile, immunotherapy is a promising treatment option for various cancers, and it has improved the quality of life of certain OC patients (2). However, immunotherapy in OC still faces challenges, such as drug resistance and the lack of preoperative non-invasive predictive tools (3).

Hypoxia impacts the tumor microenvironment (TME) (4), angiogenesis, immunosuppression, and immune evasion (5). The hypoxic microenvironment regulates carcinogenesis, radiotherapy, and chemotherapy resistance (6). Based on the above evidence, a positive response to immunotherapy may depend on immune regulation within the TME. In recent years, this theory has been proven by a series of fundamental research. For example, intratumor tissue-resident memory T cells (T_RM_) were found to express PD-1 and LAG-3, and the triggering of inhibitory receptors may lead to dysfunction that may limit the effectiveness of T_RM_ in inhibiting tumor growth (7). The attenuation of NRF1 degradation in hypoxic circumstances may impede tumor-associated macrophage polarization (8). Therefore, a comprehensive analysis of immunological characteristics due to hypoxia is a priority to improve treatment with immune checkpoint inhibitors (ICIs).

At present, a large number of studies have revealed the genesis of cancer through omics analysis. In lung cancer, key genes for disease progression were identified by various bioinformatics methods (9). Interestingly, cancer cell lines can also be identified by the incremental feature selection method (10). For OC, the ceRNA network was constructed, and novel insights of the regulatory mechanisms among mRNAs, lncRNAs, and miRNAs were provided (11). However, in most omics analyses, these studies did not focus on the combination of imaging data and sequencing data. Computed tomography (CT) is part of the standard of treatment and is used as a “road map” to guide debulking surgery and assess chemotherapy response in patients with OC (12). CT imaging-based radiomics allows for the translation of images into thousands of features followed by subsequent model building to improve prognostic prediction (13). Radiogenomics is a new cross-disciplinary research combining radiomics with genomics (14). In kidney cancer, it was shown that VHL mutations are significantly associated with well-defined tumor margins and nodular tumor enhancement (15). T2-derived texture metrics from the whole-tumor are used to assess response in therapy (16). Interestingly, radiogenomics can identify the landscape of m6A methylation modification in bladder cancer (17). Because of the intratumor heterogeneity in advanced ovarian cancer with peritoneal carcinomatosis, methods for assessing tumor heterogeneity using radiogenomics are needed to analyze whole-tumor heterogeneity rather than single biopsy sampling (18).

Hence, there have been many studies focusing on radiogenomics in ovarian cancer in recent years, but they mainly focused on the prediction of Classification of Ovarian Cancer (CLOVAR) (19) and BRCA mutations (20). Thus, we aimed to develop a radiogenomics approach to reveal the hypoxia pattern and immunological characteristics of patients with OC.

In this research, we collected the genomic data of 630 OC patients and then constructed three types of subtypes using hypoxia-related genes or hypoxia pattern regulator expression. We assessed the predictive value of the hypoxia subtypes and correlated it with TME. In addition, we developed a nine-gene next-generation sequencing panel for clinical application, and it may represent different hypoxic statuses. As for radiomics, a CT imaging signature based on the nine-gene panel classification was obtained using the radiomics algorithm. In a word, our findings revealed the critical role of hypoxia in TME and immunotherapy for OC patients. Most importantly, the CT imaging-based radiogenomics signature can make non-invasive predictions prior to treatment.

## Methods

### Datasets and Data Preprocessing

**Figure 1:**
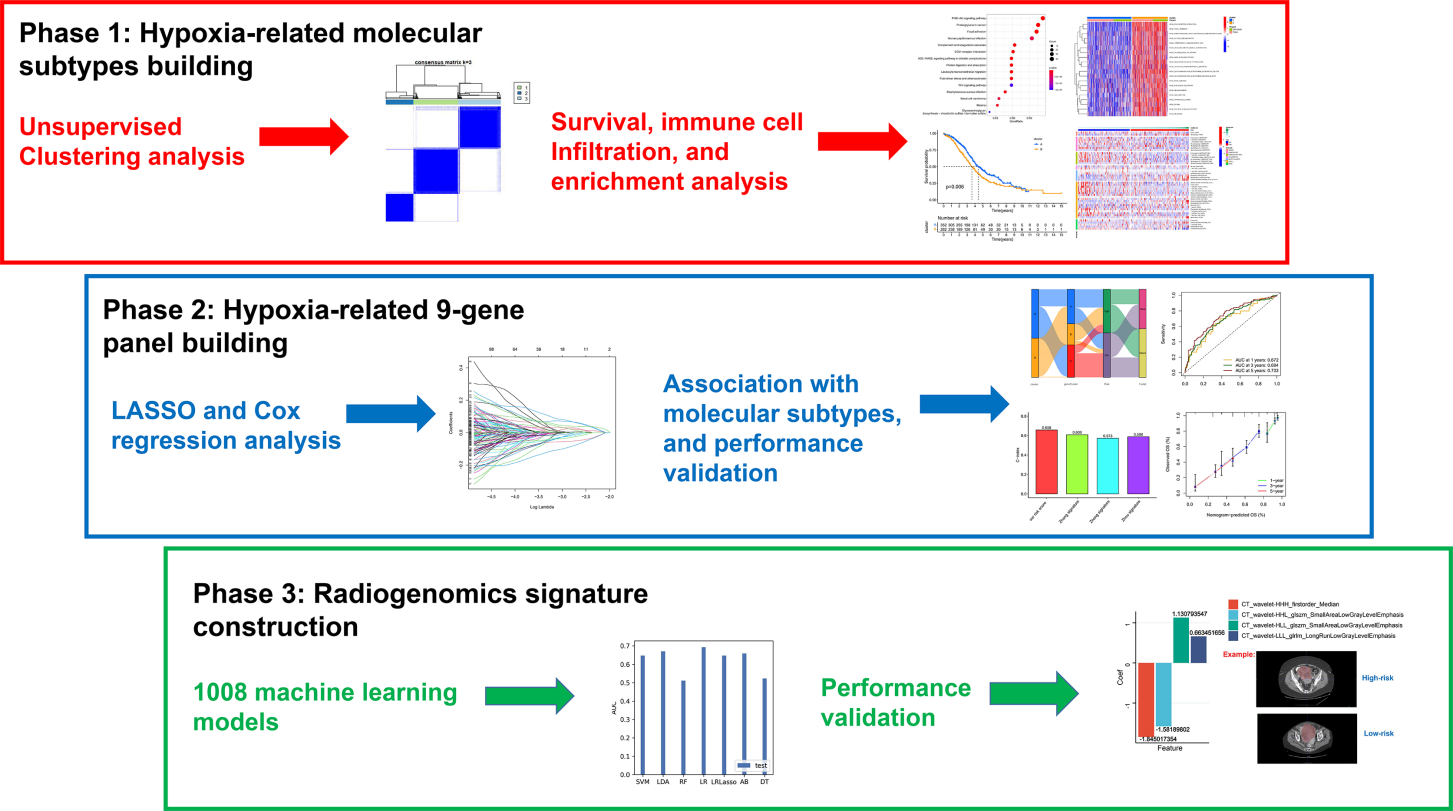
The workflow of the study.

The workflow of the study is depicted in **Figure 1**. We downloaded six samples from the GSE66894 dataset (21), namely, normoxia-cultured SKOV3 cell line samples (GSM1633848, GSM1633849, and GSM1633850) and hypoxia-cultured cell line samples (GSM1633857, GSM1633858, and GSM1633859). For hypoxia treatment, SKOV3 cells were exposed to 0.5% oxygen for 16 h. Subsequently, we used the limma package (22) for the analysis of differentially expressed genes (DEGs), and |log fold change| >1 and adj. *p*-value <0.05 were set as the thresholds (23). Meanwhile, 1,694 genes identified in previous literature were used as HRGs from the Molecular Signatures Database (MsigDB) (24). Specific hypoxia-related genes in OC were screened by the overlap of the HRGs and the DEGs. In addition, RNA sequencing profiles and clinical data of patients with OC are available from The Cancer Genome Atlas (TCGA) (25) and Gene Expression Omnibus (GEO) databases (26), and mutational data of patients with OC were obtained only from the TCGA database. We excluded samples with no survival information and those sequenced repeatedly for the same patient. Finally, 374 patients in the TCGA-OV cohort and 260 patients in GSE32062 were retained for subsequent analysis. It is worth noting that FPKM data were converted to transcripts per kilobase million (TPM) data. Batch effects between these cohorts were removed using the sva package. In addition, The mRNA stemness score (RNAss) of OC cases in TCGA was acquired from previous studies (27).

### Unsupervised Clustering Analysis

ConsensusClusterPlus package (28) was used to perform unsupervised clustering analysis for the classification of patients with OC. As for the clustering of hypoxia-related subtypes and gene-related subtypes, the parameters were set to reps = 1,000 and pitem = 0.8 based on related gene expression (29). Principal component analysis (PCA) (30) and Kaplan–Meier analysis (log-rank test) were performed to identify whether different subtypes were relatively independent in prognosis and heterogeneity.

### Immune Cell Infiltration Analysis

We simultaneously used diversified algorithms (TIMER (31), CIBERSORT (32), quanTIseq (33), MCP-counter (34), xCell (35), EPIC (36), and ssGSEA (37)] to estimate the abundances of immune cells or score of immune function in each OC sample. The ESTIMATE algorithm (38) was utilized to assess the overall state of the TME (immune score, stromal score, and tumor purity). Immune checkpoint-related gene and human leukocyte antigen (HLA) gene expressions were compared in different risk groups. In addition, the tumor immune dysfunction and exclusion (TIDE) algorithm (39) was used to assess the immunotherapy response of different patients.

### Construction and Validation of the Nine-Gene Panel

Firstly, prognostic genes (*p*-value < 0.05) were screened using univariate Cox regression analysis in all hypoxia pattern-related regulators. Next, least absolute shrinkage and selection operator (LASSO) regression analysis and multivariate Cox regression analysis (stepwise method) were used to identify genes involved in a panel. We used the appropriate *λ* and Akaike information criterion (AIC) to control robustness in the model. All the above modeling processes were carried out in the TCGA-OV cohort. The hypoxia risk score was calculated as follows:


$$\sum_{\text{i}=1}^{\text{n}}{\text{coefficient}}_{\text{i}}{*\text{expression}}_{\text{i}}$$

where coefficient is the regression coefficient in multivariate Cox regression analysis, and expression is the RNA expression of each selected gene. Considering that we used the TCGA-OV cohort as the training cohort, we calculated hypoxia risk scores in the validation cohort (GSE32062) with the same formula. Subsequently, we divided all patients into high- and low-risk groups with the median score in the TCGA cohort. Finally, PCA, ROC, Kaplan–Meier, and Cox regression analyses were used to validate the prognostic value of the nine-gene panel in each cohort.

### Comparison Between the Nine-Gene Panel and Other Signatures

Zhang et al. identified a glycolysis-related gene signature for OC patients (40). Zhou et al. identified a DNA methylation-driven gene signature (41). Moreover, Zheng et al. developed a risk stratification system based on glycolysis-related lncRNAs (42). Each signature’s risk score was determined using normalized expression values and coefficients from references. On the basis of the TCGA-OV cohort in our study (374 patients), the C-indexes of the models were estimated and compared.

### Functional Enrichment Analysis

Specific hypoxia-related genes were enriched in ClueGO of the Cytoscape software (43). The thresholds were set by default in the software. Meanwhile, we used gene set (c2.cp.kegg.v6.2.symbols) for running GSVA analysis in different hypoxia-related clusters (33). An adjusted *p*-value <0.05 was regarded as statistically significant. As for the hypoxia pattern regulators, Gene Ontology (GO) (44) and Kyoto Encyclopedia of Genes and Genomes (KEGG) (45) functional enrichment analyses were conducted. The thresholds were *p*-value <0.05 and *q*-value <0.05.

### Drug Sensitivity Analysis

The pRRophetic package worked by utilizing gene expression and drug sensitivity data in cancer cell lines, and then the models were applied to the gene expression data from primary tumor biopsies (46). We used the above method to calculate the IC50 values of different samples.

### Mutation Analysis

We used the MutSigCV package (47) to select oncogenes with higher mutation frequencies than the background and subsequently used the maftools package (48) to display the mutation waterfall figure in different groups. In addition, we used this formula (total mutation frequency/38) to estimate the tumor mutational burden (TMB) score of each sample.

### Radiomics Analysis in Computed Tomography Imaging

A total of 97 contrast-enhanced CT images of the abdomen and pelvis were selected from the Cancer Imaging Archive (TCIA) (49), which were matched with the TCGA-OV samples. The study eventually included 59 samples (inadequate image quality was excluded).

Considering the characteristics of the pelvic masses, we used arterial phase data from enhanced CT for the study. Manual segmentation was performed using ITK-SNAP in the cross-sectional layer of the largest tumor region. All patients were selected for repeat region of interest (ROI) segmentation 30 days after the initial segmentation, which was performed by different radiologists. The diversity in voxel sizes leads to variations in feature values, so for reconstruction with different voxel sizes, we used a voxel size resampling strategy to select reproducible image features: spline interpolation resamples all images to the same 1 × 1 mm pixel size. In addition, the voxel intensities within the ROI are discretized into a limited intensity range of 64 bins. Ultimately, we extracted 806 radiomics features from the ROI of each OC patient using PyRadiomics (V 2.0.0) (50). Original texture features were extracted from the texture features, shape-based features, gray-level co-occurrence matrix features, gray-level run-length matrix features, gray-level size zone matrix features, and gray-level difference matrix features. The repeatability of the retrieved characteristics from the two radiologists was validated using the intraclass correlation coefficient (ICC). In the succeeding studies, only characteristics with an interreader ICC >0.75 were included. Using nine-gene panel as a classifier, we established radiogenomics prediction models based on radiomics features from the ROI. We randomly selected 40 cases as the training dataset, and the remaining 19 cases were used as the testing dataset. The best AUC value in the testing dataset was utilized as the selection criterion to identify the best technique to develop the final model after we employed different dimensionality reductions and machine learning approaches for imaging genomics model construction. The above modeling processes were implemented using FeAture Explorer Pro (V 0.4.4) (51).

### Statistical Analysis

All statistical analyses were performed using the R software (v.4.0.1) and Python (v.3.7.6). Detailed statistical methods for transcriptome data processing are covered in the above section. *p <*0.05 was considered statistically significant.

## Results

### Specific Hypoxia-Related Genes in OC

**Figure 2:**
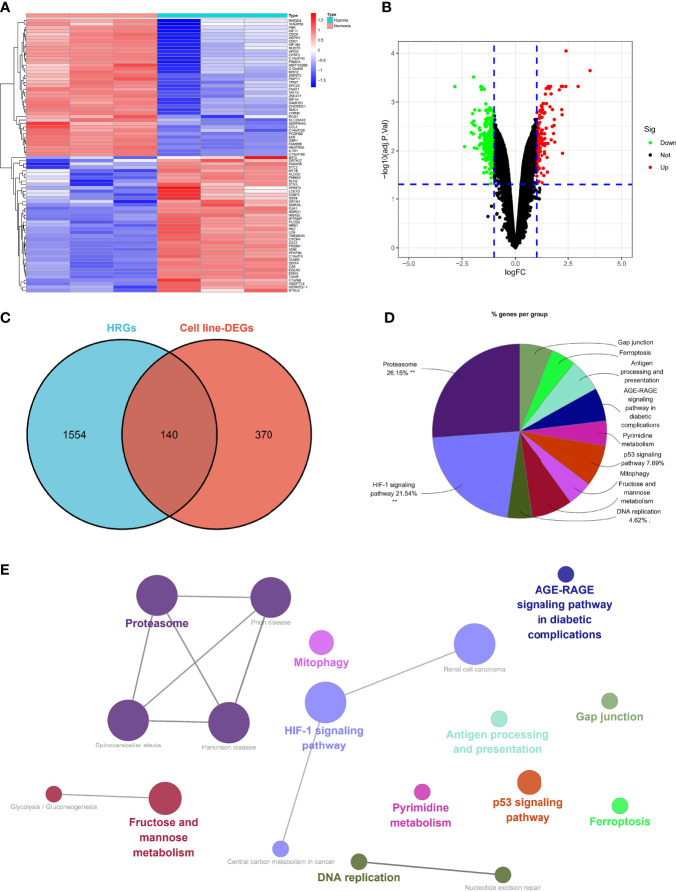
Identification of sHRGs in ovarian cancer (OC). Heat map **(A)** and volcano plot **(B)** of differentially expressed genes (DEGs) in SKOV3 cell lines. **(C)** Venn diagram of hallmark gene sets from the MSigDB and DEGs. **(D)** Pie chart of ClueGO analysis. **(E)** Network diagram of ClueGO analysis.

Five hundred and ten DEGs were identified in the normoxia- and hypoxia-cultured OC cell lines (**Figures 2A, B** and **Supplementary File 1**). Subsequently, we overlapped the hallmark gene sets and the DEGs, and a Venn diagram showed 140 shared genes as sHRGs (**Figure 2C**). We performed ClueGO analysis in Cytoscape software to verify whether 140 sHRGs were associated with hypoxia-related metabolic processes. Not surprisingly, the results showed that sHRGs were mainly enriched in the proteasome and classical HIF-1 signaling pathways (**Figures 2D, E**).

Our data showed that 140 sHRGs were identified in cell lines and associated with hypoxia-related metabolic processes.

### Characteristics of sHRG-Mediated Hypoxia Patterns

**Figure 3:**
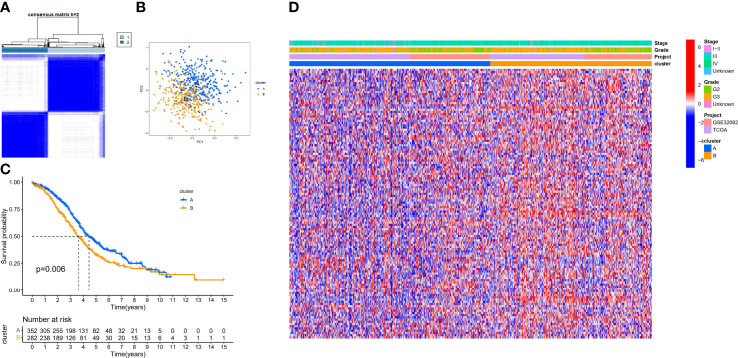
The different hypoxia patterns in patients with OC. **(A)** Heat map of unsupervised clustering analysis. **(B)** Principal component analysis (PCA) analysis of different hypoxia patterns. **(C)** Kaplan–Meier analysis of overall survival time in different hypoxia patterns. **(D)** Heat map of the distribution of clinicopathological characteristics and two hypoxia patterns.

**Figure 4:**
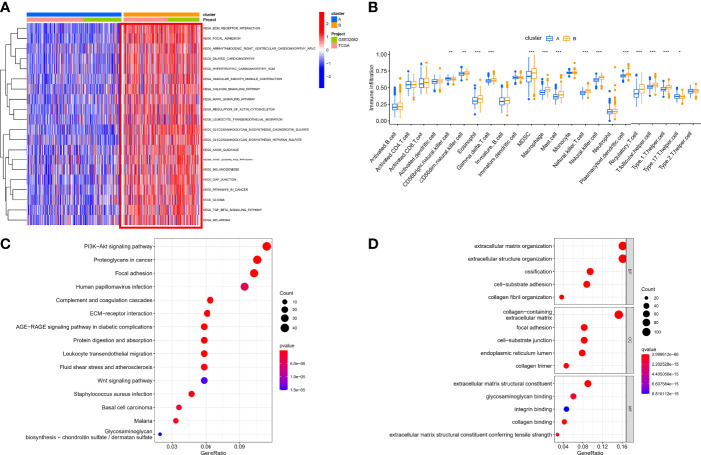
The immunological and biological characteristics in different hypoxia patterns. **(A)** GSVA analysis in different hypoxia patterns using kegg.v7.4 gene sets. **(B)** Box plot of immune cells in different hypoxia patterns. **(C)** Bubble plot of KEGG enrichment analysis. **(D)** Bubble plot of GO enrichment analysis. *p < 0.05, **p < 0.01, ***p < 0.001.

Based on the expression of 140 sHRGs, patients with OC were classified into two hypoxia patterns using unsupervised clustering analysis, namely, hypoxia-cluster-A (352 patients) and hypoxia-cluster-B (282 patients) (**Figure 3A**). PCA analysis revealed that the above two patterns were relatively independent (**Figure 3B**). Survival analysis showed that hypoxia-cluster-B had the worst prognosis (**Figure 3C**). Moreover, we also plotted heat maps to show the distribution of clinicopathological characteristics and hypoxia patterns (**Figure 3D**). Subsequently, GSVA and ssGSEA algorithms focused on biological processes and immune microenvironment between the different hypoxia patterns. The results showed that hypoxia-cluster-B was significantly upregulated in most pathways and showed immune activation characteristics, including the MAPK signaling pathway, Wnt signaling pathway, ECM–receptor interaction, MDSC, and NK cells (**Figures 4A, B**). Therefore, it is reasonable to assume that hypoxia-cluster-B showed an immune-inflamed tumor phenotype, and they may be the most responsive to immunotherapy. If immunotherapy is applied routinely, it will prolong the survival time in hypoxia-cluster-B. Although the hypoxia patterns could differentiate clinical outcomes in patients, the underlying regulators in these patterns are unknown. Hence, we identified DEGs in different hypoxia patterns (**Supplementary File 2**). The enrichment analysis of 770 regulators in different hypoxia patterns was carried out in GO and KEGG analyses (**Figures 4C, D**). Interestingly, the PI3K–Akt signaling pathway was significantly activated, which may suggest that it may play a key role in hypoxia-related metabolic processes in OC.

Our data showed that two hypoxia patterns were identified in the meta cohort, and hypoxia patterns suggested different immune phenotypes.

### Identification of Hypoxia Pattern-Related Regulator Subtypes

**Figure 5:**
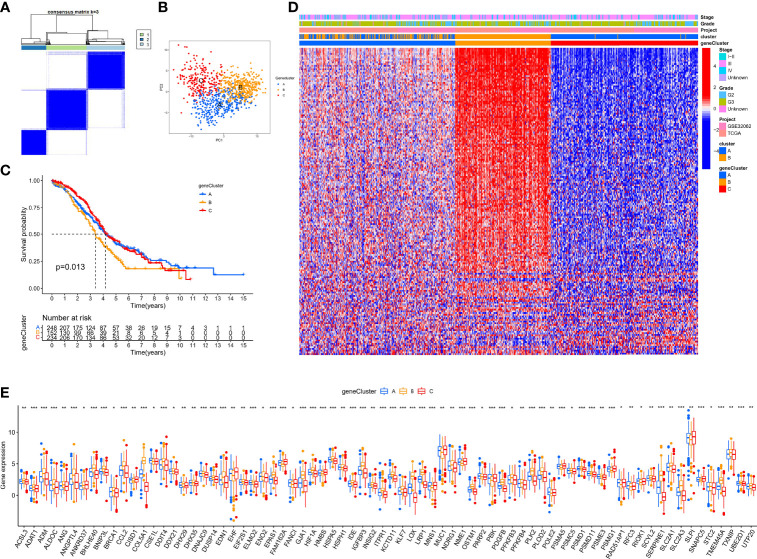
Hypoxia pattern-related regulator subtypes. **(A)** Heat map of unsupervised clustering analysis. **(B)** PCA analysis of different gene subtypes. **(C)** Kaplan–Meier analysis of overall survival time in different gene subtypes. **(D)** Heat map of distribution of clinicopathological characteristics and three gene subtypes. **(E)** Box plot of 140 sHRGs in three subtypes. *p < 0.05, **p < 0.01, ***p < 0.001.

In the above section, we screened out 770 differential expression genes in different hypoxia patterns to focus on their potential OC mechanisms. Based on the expression of 770 regulators, patients were classified into three subtypes using unsupervised clustering analysis, namely, gene-cluster-A (248 patients), gene-cluster-B (152 patients), and gene-cluster-C (234 patients) (**Figure 5A**). PCA analysis revealed that the above three subtypes were relatively independent (**Figure 5B**). Survival analysis showed that gene-cluster-B had the worst prognosis (**Figure 5C**). Interestingly, the heat map showed that most regulators were significantly upregulated in gene-cluster-B than in the other subtypes (**Figure 5D**). In addition, we also compared the differential expression of 140 sHRGs in the three subtypes, and excitingly, all sHRGs were significantly different (**Figure 5E**).

Our data showed that hypoxia pattern-related regulator subtypes suggested another perspective on their critical regulating role on the hypoxic microenvironment.

### Identification of the Hypoxia Risk Score for Each Patient With OC

**Figure 6:**
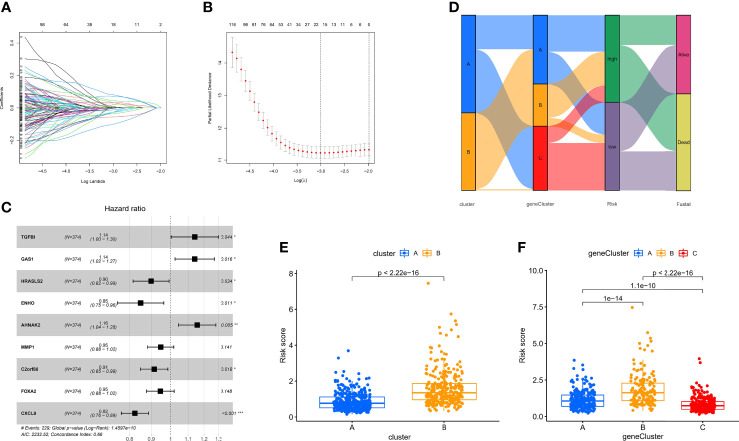
Identification of the nine-gene next-generation sequencing panel. **(A, B)** Determination of the number of regulators using LASSO analysis. **(C)** Forest plot of multivariate Cox regression analysis. **(D)** Sankey diagram of the three types of subtypes. **(E)** Analysis of differences in hypoxia risk score of different hypoxia patterns. **(F)** Analysis of differences in hypoxia risk score of different hypoxia pattern-related regulator subtypes.

Although the hypoxia patterns or regulator subtypes can predict differences in survival and immune characteristics, molecular subtypes were studied based on patient populations. The above method cannot accurately predict the hypoxia risk status of each patient, so we evaluated individual patients based on the RNA expression of the above regulators for clinical application with the risk score. Firstly, regulators with *p <*0.05 from the univariate Cox regression analysis (TCGA-OV cohort) were included in the LASSO regression analysis (**Supplementary File 3**). Subsequently, redundant regulators were removed by LASSO regression (**Figures 6A, B**), and correlation coefficients were determined by multivariate Cox regression analysis (stepwise method) (**Figure 6C**). Finally, we developed a nine-gene panel calculating risk scores, namely, TGFBI, GAS1, HRASLS2, ENHO, AHNAK2, MMP1, C2orf88, FOXA2, and CXCL9. The formula for calculating the hypoxia risk score is as follows: hypoxia risk score = (0.132009353 × expression level of TGFBI) + (0.131635755 × expression level of GAS1) + (−0.106191762 × expression level of HRASLS2) + (−0.163100133 × expression level of ENHO) + (0.145369988 × expression level of AHNAK2) + (−0.053663201 × expression level of MMP1) + (−0.089183891 × expression level of C2orf88) + (−0.055649255 × expression level of FOXA2) + (−0.194630892 × expression level of CXCL9).

Considering that we used the TCGA-OV cohort as the training cohort, we also calculated patients’ risk scores in the validation cohort (GSE32062) with the same formula. Subsequently, we divided all patients with OC into high- and low-risk groups with the median score in the training cohort. To explore the relationship between the three subtypes, namely, hypoxia patterns, pattern-related regulator subtypes, and hypoxia risk group, we visualized the relationship using the Sankey diagram (**Figure 6D**). The results showed that most patients with poor prognosis in molecular subtypes were closely related to patients in the high-risk group. In addition, the box plot confirmed our results that hypoxia-cluster-B and gene-cluster-B had higher hypoxia risk scores (**Figures 6E, F**).

Our data showed that the nine-gene next-generation sequencing panel may represent different hypoxic statuses and be more convenient for clinical application.

### Prognostic Value of Hypoxia Risk Score

**Figure 7:**
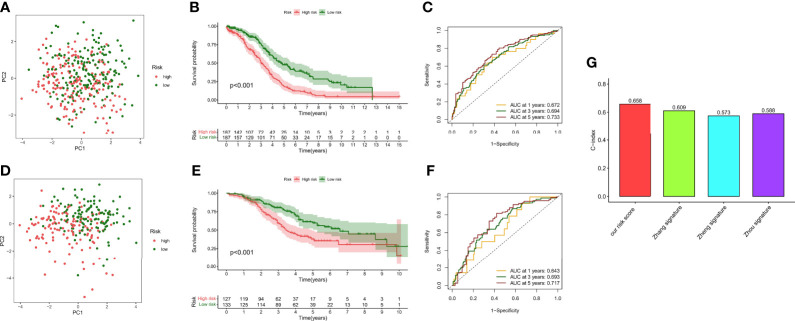
Survival analysis and model comparison of the hypoxia risk score. **(A)** PCA analysis in the TCGA cohort. **(B)** Kaplan–Meier analysis of different risk groups in the TCGA cohort. **(C)** ROC curve of the 1-, 3-, and 5-year survival prediction in the TCGA cohort. **(D)** PCA analysis in the GEO cohort. **(E)** Kaplan–Meier analysis of different risk groups in the GEO cohort. **(F)** ROC curve of the 1-, 3-, and 5-year survival prediction in the GEO cohort. **(G)** C-index of different risk scores.

Although a small portion of the sample was mixed, PCA analysis demonstrated that hypoxic risk scores had a potential classification ability for the TCGA cohort (**Figure 7A**) and the GEO cohort (**Figure 7D**). Kaplan–Meier analysis showed that survival time was significantly shorter in the high-risk group than in the low-risk group (**Figures 7B, E**), which indicated that hypoxia risk score has an excellent predictive value. Meanwhile, the AUC values of the TCGA cohort (**Figure 7C**) and the GEO cohort (**Figure 7F**) at 1, 3, and 5 years reached 0.672, 0.694, and 0.733 and 0.643, 0.693, and 0.717, respectively. To highlight the predictive value of the hypoxia score, we compared another risk score from references, such as glycolysis genes, DNA methylation-driven genes, and glycolysis-related lncRNAs. In 374 patients from the TCGA cohort, the C-index value showed that hypoxia risk score had the most robust predictive performance (**Figure 7G**). In addition, we performed univariate and multivariate Cox regression analyses of the hypoxia risk score and clinical characteristics in different cohorts. The results showed that hypoxia risk score is an independent prognostic factor in the TCGA cohort (**Supplementary Figures 1A, B**) and the GEO cohort (**Supplementary Figures 1C, D**). Finally, we plotted a nomogram based on risk group and another significant factor in multivariate Cox regression analysis (**Supplementary Figure 1E**). The calibration curve showed that the prediction curves are close to the standard curve in the TCGA cohort (**Supplementary Figure 1F**) and the GEO cohort (**Supplementary Figure 1G**).

Our data showed that hypoxia risk score had an excellent survival prediction ability.

### Immunological Characteristics of Hypoxia Risk Score

**Figure 8:**
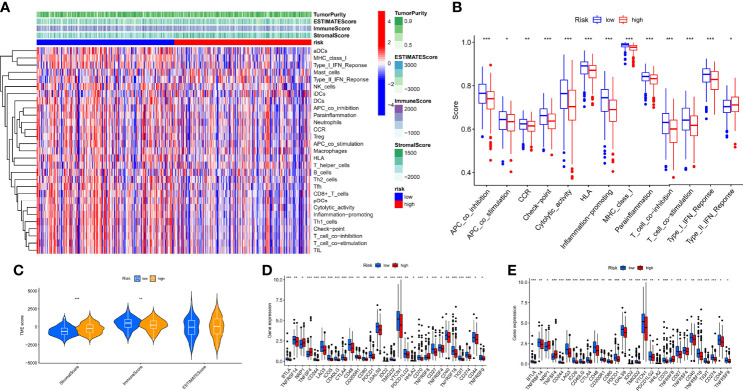
Characteristics of the immune microenvironment in different risk groups. **(A)** Heat map of the result of ssGSEA and ESTIMATE algorithm. **(B)** Analysis of differences in the immune function of different risk groups. **(C)** Analysis of differences in TME score of different risk groups. **(D)** Analysis of differences in immune checkpoint mRNA expression of different risk groups. **(E)** Analysis of differences in HLA mRNA expression of different risk groups. *p < 0.05, **p < 0.01, ***p < 0.001.

To comprehensively explore the relationship between different risk groups and immune cell infiltration, we explored immune cell infiltration based on the six algorithms. The heat map showed immune cells with differential distribution in different algorithms (**Supplementary Figure 2**). Interestingly, the low-risk group had more abundant levels of antitumor immune cell infiltration, such as NK cells, CD4^+^ T cells, CD8^+^ T cells, macrophages, and mast cells. Not all patients in the TCGA-OV cohort received immunotherapy; hence, we evaluated the ability of hypoxia risk score to predict immunotherapy response and survival in the cohort treated with anti-PD-L1 [IMvigor (52)]. As with the TCGA-OV cohort, patients with high hypoxia risk score had worse OS in the IMvigor cohort (**Supplementary Figure 3A**). Excitingly, in the complete remission (CR) or partial response (PR) subgroup, patients typically had a lower hypoxia risk score (**Supplementary Figure 3B**). We used the ssGSEA algorithm to explore changes in immune function and the ESTIMATE algorithm to explore changes in the immune microenvironment (**Figure 8A**). We found immune function in a more active state, higher immune score, and lower stromal score in the low-risk group (**Figures 8B, C**). In addition, we also explored immune checkpoint and HLA mRNA expression in different risk groups. Most of the HLA and immune checkpoints were upregulated in the low-risk group, such as PDCD1, CTLA4, CD274, HLA-A, and HLA-F (**Figures 8D, E**).

**Figure 9:**
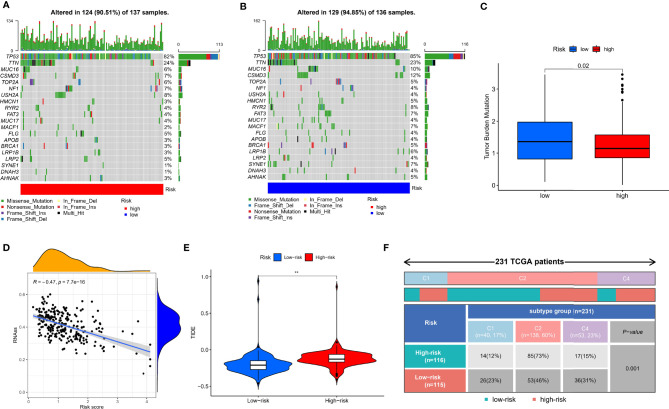
Mutation, TIDE, and stemness characteristics in different risk groups. **(A)** Frequency of somatic mutations in the high-risk group. **(B)** Frequency of somatic mutations in the low-risk group. **(C)** Analysis of differences in TMB score of different risk groups. **(D)** Correlation analysis of hypoxia risk score with stemness score. **(E)** Analysis of differences in TIDE score of different risk groups. **(F)** The distribution of immunological subtypes in different risk groups. **p < 0.001.

Given that TMB and immunotherapy are strongly associated in a study (53), we explored somatic mutation characteristics and TMB status in different risk groups. Among the different risk groups, TP53, TTN, and MUC16 were shared mutated genes (**Figures 9A, B**). In the low-risk group, the samples had a higher rate of mutation (94.85% vs. 90.51%). Notably, the box plot showed that the low-risk group had a higher TMB score (**Figure 9C**). The cancer stemness theory posits that stemness scores are a response factor in immunotherapy (54). We found that as the hypoxia risk score increased, the stemness score decreased (**Figure 9D**). Moreover, the TIDE algorithm was used to evaluate the response to immunotherapy. The results showed that the low-risk group had a lower TIDE score, as we predicted in the IMvigor cohort, representing the possibility that the low-risk group had a better response to immunotherapy (**Figure 9E**). Thorsson et al. developed six immune subtypes across more than 10,000 tumor samples comprising 33 diverse cancer types (55). Of these, three immune subtypes can be annotated in the TCGA-OV cohort (231 patients), namely, Immune C1, Immune C2, and Immune C4. There is no doubt that our risk groupings were distributed differently among the different immunophenotypes (**Figure 9F**).

Our data showed new insights into the mechanisms underlying tumor hypoxia risk score and immunotherapy.

### The Role of Hypoxia Risk Score in Chemotherapy

The IC50 values of six common chemotherapeutic medicines were quantified in OC patients, namely, bleomycin (**Supplementary Figure 4A**), cisplatin (**Supplementary Figure 4B**), paclitaxel (**Supplementary Figure 4C**), docetaxel (**Supplementary Figure 4D**), etoposide (**Supplementary Figure 4E**), and gemcitabine (**Supplementary Figure 4F**). In detail, the IC50 levels of bleomycin and docetaxel were significantly higher in the low-risk group. In contrast, the IC50 levels of paclitaxel were significantly higher in the high-risk group.

Our data indicated that the low-risk group was more sensitive to paclitaxel, while the high-risk group was more sensitive to bleomycin and docetaxel.

### Construction of Optimal Radiomics Signatures

**Figure 10:**
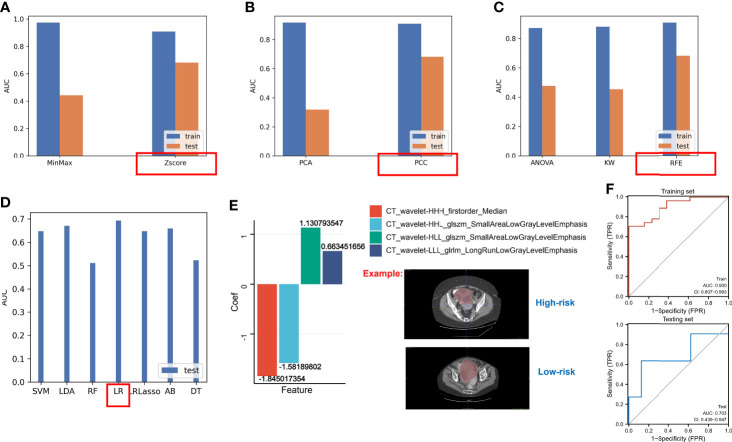
The AUC values of different methods. **(A)** The AUC values of methods for normalization. **(B)** The AUC values of methods for feature preprocessing. **(C)** The AUC values of methods for dimensionality reduction. **(D)** The AUC values of methods for generating the final signature. **(E)** LR regression coefficients and features. **(F)** The AUC values of the testing set and the training set based on the optimal radiomics signature.

Based on the above results, the hypoxia risk score based on the nine-gene next-generation sequencing panel had a possibility for clinical application, but the method is still invasive. Hence, we used the radiomics approach to match with different risk groups. We selected 40 cases as the training set and another 19 cases as the independent testing set. Using the constructed different risk groups (high-risk and low-risk) as a classifier, we extracted the radiomics features from these CT images for the established radiogenomics signature. A total of 1,008 models were constructed by combining different methods (**Supplementary File 4**). We found that the combination of the following methods had better AUC values: the *Z*-score method for normalization (**Figure 10A**), the PCC method for feature preprocessing (**Figure 10B**), the RFE method for dimensionality reduction (**Figure 10C**), and the logistic regression method for calculating coefficient (**Figure 10D**). Finally, we obtained the following four features and coefficients for constructing the optimal radiomics signatures (**Figure 10E**): radiomics score = (−1.845017354 × CT_wavelet-HHH_firstorder_Median) + (−1.58189802 × CT_wavelet-HHL_glszm_SmallAreaLowGrayLevelEmphasis) + (1.130793547 × CT_wavelet-HLL_glszm_SmallAreaLowGrayLevelEmphasis) + (0.663451656 × CT_wavelet-LLL_glrlm_LongRunLowGrayLevelEmphasis). Using the above radiomics signature, the AUC values of the training set and the test set were 0.900 and 703, respectively (**Figure 10F**).

Our data showed that a novel non-invasive approach based on CT biomarkers may enable the pretreatment prediction of hypoxia risk in patients with OC.

## Discussion

There is growing evidence that the hypoxia microenvironment plays a key role in immune response and tumorigenesis on the basis of dysregulated expression of hypoxia-related genes. Most previous studies focused on single regulators about hypoxia in OC. For example, hypoxia-inducible factor-1α (HIF-1α) has been proven to play an important role in promoting OC chemoresistance, tumor metastasis, and immunosuppression (56). The high expression of maspin induced by hypoxia might be associated with a poor prognosis of ovarian clear cell carcinoma (57). More importantly, although some researchers have identified hypoxia-related genetic signatures to improve the prognosis of patients with ovarian cancer (58, 59), they neglected that specific hypoxia-related genes in OC should be analyzed to construct a risk signature. Hence, we performed the identification of specific hypoxia regulators in OC based on cell lines treated with different oxygen conditions. Subsequently, we established different hypoxia patterns (hypoxia-cluster-A and hypoxia-cluster-B) and identified regulators that may influence different hypoxia patterns. Moreover, we also established different gene subtypes (gene-cluster-A and gene-cluster-B) based on the expression of regulators. In immune analysis, we revealed that hypoxia-cluster-B and gene-cluster-B correspond to the immune-inflamed phenotype, which contains many antigen-presenting cells that activate an adaptive immune response. However, hypoxia-cluster-A and gene-cluster-A correspond to the immune-excluded phenotype (60). To date, immunotherapy outcomes in OC have been disappointing, likely due to the highly immunosuppressive TME, low TMB, and low checkpoint expression in patients with OC (61). Therefore, our study provides a novel understanding of the OC microenvironment based on hypoxia. If immunotherapy is applied routinely, it will prolong the survival time in hypoxia-cluster-B and gene-cluster-B. Hence, these findings will improve the future application of precise personalized therapy for OC.

Although hypoxia patterns could differentiate clinical outcomes and immune perturbations in OC patients, the underlying regulators in different patterns are unknown. We found significantly altered pathways in the enrichment analysis, including the MAPK signaling pathway, Wnt signaling pathway, ECM–receptor interaction, and PI3K–Akt signaling pathway. As a classic cancer pathway, these pathways have been widely reported in ovarian cancer (62, 63). However, our findings gave researchers a new direction in the future: how does hypoxia interfere with TME in OC through the classic cancer pathways?

Although the hypoxia patterns can predict differences in survival and immune characteristics, molecular subtypes were studied based on populations. The above method cannot accurately predict the hypoxia risk status of each patient, so we used the RNA expression of nine hypoxia pattern-associated regulators for clinical application with hypoxia risk score. Most patients with poor prognosis in molecular subtypes were closely related to patients in the high-risk group, and hypoxia-cluster-B and gene-cluster-B had higher hypoxia risk score. Of the nine genes, some of them were shown to be involved in the regulation of biological functions in ovarian cancer, such as TGFBI which is involved in polyploid cell formation and in response to paclitaxel (64); extracellular vesicles carrying the MMP1 mRNA promoted peritoneal metastasis of ovarian cancer (65). The FOXA2 protein was mainly positively expressed in the nucleoplasm of OC cells and was associated with FIGO staging and lymph node metastasis (66). Further analysis revealed that hypoxia risk score could be used not only to predict the prognosis of patients with OC but also to accurately distinguish different immunological characteristics. In addition, HLA mRNA expression, immune checkpoint mRNA expression, TMB, and stemness score were significantly correlated with hypoxia risk score, indicating the ability of the risk score to assess the effectiveness of immunotherapy. We found that the low-risk group was more sensitive to paclitaxel, while the high-risk group was more sensitive to bleomycin and docetaxel. It follows that the quantitative model can define the hypoxia status of each sample. Thus, these results validate that the hypoxia-related model can be conveniently used for clinical assessment.

>However, genomic models are invasive; therefore, we developed a convenient method to predict hypoxic subtypes based on CT imaging in our study. Although the sample size of OC in the TCIA database is limited, we found the following combinations of methods with better AUC values: the *Z*-score method for normalization, the PCC method for feature preprocessing, the RFE method for dimensionality reduction, and the logistic regression method for calculating coefficient. Using the above radiomics signature, the AUC values of the training set and the test set were 0.900 and 703, respectively. There is extensive heterogeneity at the genomic level in primary OC and peritoneal implants, and single-site biopsy sequencing clearly does not meet our requirements (67), at which point a radiogenomics approach can provide a comprehensive assessment (68).

In brief, the current studies of radiogenomics in OC are minimal and mainly plagued by time-consuming manual segmentation. However, based on current artificial intelligence (AI) research on other tumors (69), we speculate that radiogenomics in OC could be used as novel biomarkers for drug selection and assessment of immunological characterization in the future.

## Data Availability Statement

The datasets presented in this study can be found in online repositories. The names of the repository/repositories and accession number(s) can be found in the article/**Supplementary Material**.

## Author Contributions

SF and TX conceived and designed the study. YS was responsible for the materials. SF drafted the article. YS, SL, YG, XJ, and KZ revised the article critically. All authors provided their final approval of the submitted version.

## Funding

This study was supported by the National Natural Science Foundation of China (No. 82072078), Jiangsu Province Key Research and Development Project (SBE2020741118), and Postgraduate Research & Practice Innovation Program of Jiangsu Province.

## Conflict of Interest

The authors declare that the research was conducted in the absence of any commercial or financial relationships that could be construed as a potential conflict of interest.

## Publisher’s Note

All claims expressed in this article are solely those of the authors and do not necessarily represent those of their affiliated organizations, or those of the publisher, the editors and the reviewers. Any product that may be evaluated in this article, or claim that may be made by its manufacturer, is not guaranteed or endorsed by the publisher.

## Supplementary Material

Supplementary Figure 1 Identification of independent prognostic value and Nomogram. **(A)** Forest plot of univariate Cox regression analysis in the TCGA cohort. **(B)** Forest plot of multivariate Cox regression analysis in the TCGA cohort. **(C)** Forest plot of univariate Cox regression analysis in the GEO cohort. **(D)** Forest plot of multivariate Cox regression analysis in the GEO cohort. **(E)** Nomogram. **(F)** Calibration curve of nomogram for OS prediction at 1 year, 3 years, and 5-years in the TCGA cohort. **(G)** Calibration curve of nomogram for OS prediction at 1 year, 3 years, and 5-years in the GEO cohort.

Supplementary Figure 2 Heat map of different types of immune cells based on 6 algorithms.

Supplementary Figure 3 Predictive value of hypoxia risk score in the immunotherapy cohort. **(A)** Kaplan-Meier analysis of IMvigor cohort. **(B)** Analysis of differences in hypoxia risk score of different response.

Supplementary Figure 4 Drug sensitivity analysis in different risk groups. **(A)** IC50 levels of Bleomycin. **(B)** IC50 levels of Cisplatin. **(C)** IC50 levels of Paclitaxel. **(D)** IC50 levels of Docetaxel. **(E)** IC50 levels of Etoposide. **(F)** IC50 levels of Gemcitabine.

Supplementary File 1 DEGs in normoxia and hypoxia cultured cell lines.

Supplementary File 2 DEGs in different hypoxia patterns.

Supplementary File 3 The results of univariate Cox regression analysis in 770 reguators.

Supplementary File 4 The results of 1008 radiomics signatures in training and testing sets.

## Abbreviations

OC, ovarian cancer; sHRGs, specific hypoxia-related genes; AUC, area under the curve; TME, tumor microenvironment; ICIs, immune checkpoint inhibitors; CT, computed tomography; CLOVAR, Classification of Ovarian Cancer; DEGs, differentially expressed genes; MsigDB, Molecular Signatures Database; TCGA, The Cancer Genome Atlas; PCA, principal component analysis; TIDE, tumor immune dysfunction and exclusion; TCIA, The Cancer Imaging Archive; ROI, region of interest.
