# Supplementary figures and images for: Computed Tomography Imaging-Based Radiogenomics Analysis Reveals Hypoxia Patterns and Immunological Characteristics in Ovarian Cancer
Source: Front Immunol. 2022 Mar 28;13:868067. doi: 10.3389/fimmu.2022.868067 (PMC8995567; doi:10.3389/fimmu.2022.868067)

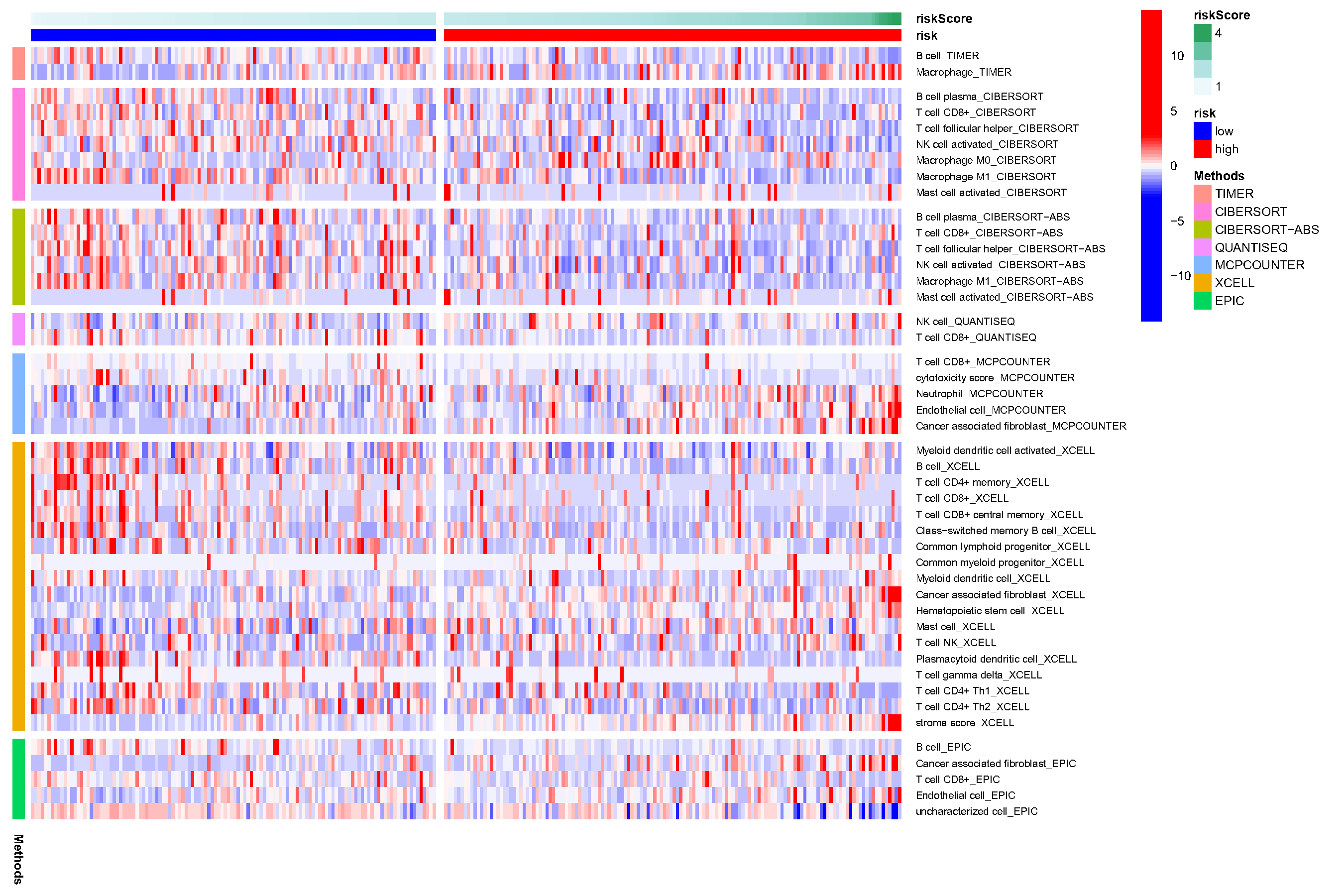

Supplement: Supplementary Figure 2 — Heat map of different types of immune cells based on 6 algorithms. [file Image_2.tiff]

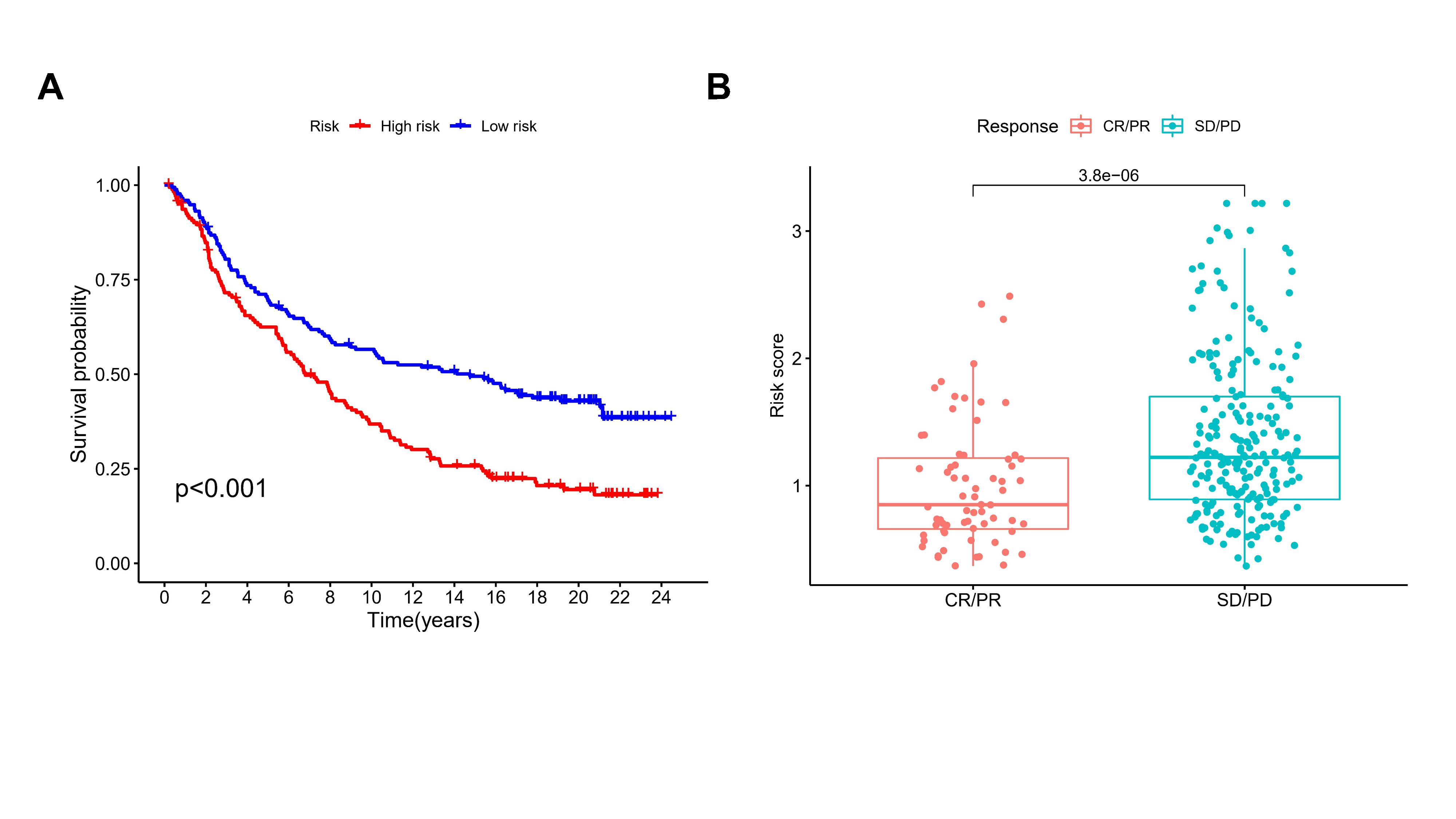

Supplement: Supplementary Figure 3 — Predictive value of hypoxia risk score in the immunotherapy cohort. (A) Kaplan-Meier analysis of IMvigor cohort. (B) Analysis of differences in hypoxia risk score of different response. [file Image_3.tiff]

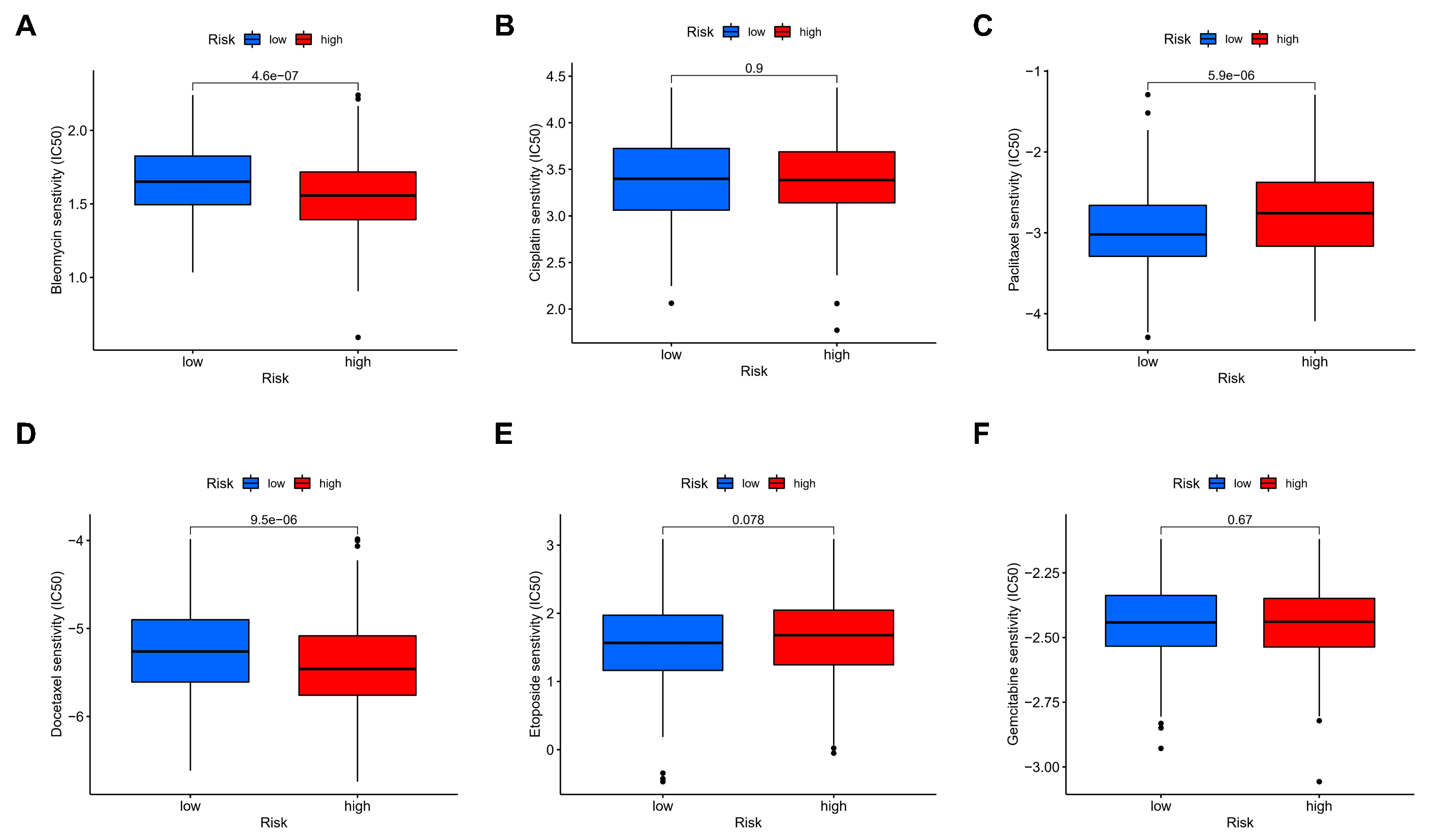

Supplement: Supplementary Figure 4 — Drug sensitivity analysis in different risk groups. (A) IC50 levels of Bleomycin. (B) IC50 levels of Cisplatin. (C) IC50 levels of Paclitaxel. (D) IC50 levels of Docetaxel. (E) IC50 levels of Etoposide. (F) IC50 levels of Gemcitabine. [file Image_4.tiff]
